# Breaking challenges: queer perspectives on solutions to establish inclusive sexual-reproductive healthcare in Gauteng Province, South Africa

**DOI:** 10.3389/fsoc.2024.1406265

**Published:** 2024-08-06

**Authors:** Raikane James Seretlo, Mathildah Mpata Mokgatle, Hanlie Smuts

**Affiliations:** ^1^Department of Public Health, Sefako Makgatho Health Sciences University, Tshwane, South Africa; ^2^Department of Informatics, University of Pretoria, Tshwane, South Africa

**Keywords:** challenges, queer, perspectives, inclusive, sexual-reproductive healthcare, Gauteng Province, South Africa, solutions

## Abstract

**Introduction:**

In South Africa’s diverse population, queer voices emerge as change agents, proposing new and critical solutions to reform sexual and reproductive healthcare services in the pursuit of inclusivity and equality. Our research aimed to explore and describe queer individuals’ perceptions and solutions for promoting sexual-reproductive healthcare services and needs (SRHSN) in Gauteng Province, South Africa.

**Methodology:**

An explorative-descriptive study was performed for this research. A total of 22 queer individuals was recruited using respondent-driven sampling (RDS) within a queer-inclusive non-governmental organization (NGO) clinic in Gauteng Province, South Africa. Semi-structured interviews and an interview guide were utilized to collect data using English. Thematic content analysis was applied using NVivo 14.

**Results:**

Four main themes and related sub-themes were revealed from the study including the creation of healthcare equity for queer individuals, empowering and supporting healthcare providers (HCPs) to enhance skills, knowledge, and expertise, raising awareness on queer-related issues, and different stakeholders’ involvement.

**Conclusion:**

As we navigate South Africa’s complex landscape of SRHSN for queer individuals, the crucial opinions and solutions offered by queer people not only challenge the status quo but also reveal an approach to a better, more equitable, empathetic, and accommodating healthcare system for everyone. Future studies should ensure the inclusiveness of queer individuals with the interest of improving their health not just for research outputs.

## 1 Introduction

South African queer individuals continue to experience challenges of access to healthcare services due to unfriendly HCPs, non-inclusive healthcare facilities, and poorly equipped infrastructures (Mueller, 2016; Mkhize and Maharaj, 2021). This is regardless of the South African constitution’s promise that every person has a right to access healthcare services including reproductive healthcare (Constitution of the Republic of South Africa, 1996). Therefore, the question that South African policymakers and external health stakeholders should be asking is whether they are truly promoting inclusivity and diversity in sexual and reproductive healthcare by incorporating queer health needs through their perspectives during policy development, or if they are disregarding a vital voice in the construction of SRHSN.

Queer is an inclusive expression that includes not only lesbian and gay people but also anybody who feels discriminated against because of their sexual behaviors and practices (health Do, 2017). In this article, the term queer will be used for someone who is not heterosexual or cisgender. South Africa introduced and implemented a policy called South African National LGBTI HIV Plan, 2017–2022 (DoH), 2020 to enhance queer people’s health by ensuring that everyone has access to comprehensive health services; to empower queer people to conquer economic and social obstacles, fostering psychosocial wellbeing through counseling and prevention support; to ensure queer human rights are safeguarded against the public and healthcare providers (HCPs); and to evaluate whether or not segregation is efficient (health Do, 2017).

However, based on the different South African studies, queer individuals continue to experience prejudice, judgment, discriminatory comments, and healthcare disparities (Luvuno et al., 2017; Spencer et al., 2017; Wingo et al., 2018; Luvuno et al., 2019; Mkhize and Maharaj, 2020, 2021). One of the most significant barriers to South African queer people receiving and making use of healthcare services is an inadequate level of education, comprehension, and competence among healthcare practitioners concerning queer people’s healthcare needs (Müller, 2017; Kurebwa, 2022; Seretlo and Mokgatle, 2022). Furthermore, research studies have shown that heteronormative views among healthcare staff, a deficiency of training, and understanding of the queer community and supporting structural factors all serve as impediments to providing high-quality healthcare to queer individuals (Luvuno et al., 2017, 2019; Spencer et al., 2017). These difficulties faced by queer people have been noticed over the world. According to Alencar Albuquerque et al. (2016), queer people face barriers to accessing healthcare due to heteronormative beliefs enforced by health providers. As stated by Hafeez et al. (2017), queer individuals receive poor quality care because of stigma, a lack of awareness among healthcare providers, and insensitivity to their community’s specific needs. Furthermore, other research found that queer individuals are marginalized, discriminated against, and stigmatized in the public primary healthcare (PHC) and health systems, resulting in unequal access to healthcare services (Gonzales and Henning-Smith, 2017; Taşkın et al., 2020; Ross et al., 2021; Mulemfo et al., 2023).

Addressing the issues that queer individuals face is critical for reducing health risks on time. For instance, if these issues are not addressed and prioritized, there will continue to be barriers to receiving some of their SRHSN, such as condoms, lubricants, dental dams, and human immunodeficiency virus (HIV) pre-exposure prophylaxis (PrEP) (Mavhandu-Mudzusi, 2016). Queer people will continue to be marginalized and rejected and experience phobias, discrimination, despair, self-harming behaviors, and have thoughts of suicide (Wilson and Cariola, 2020). Finally, there will be a higher likelihood of certain sexually transmitted infections (STIs), such as HIV or acquired immunodeficiency syndrome (AIDS), while some queer people may still gain weight, leading to obesity and undiagnosed cancer types due to a lack of mammography or Papanicolaou test screening (Daniel et al., 2015). Fewer studies regarding the queer individual’s voices to suggest solutions that can improve their SRHSN have been conducted, as most studies focused on queer experiences in healthcare facilities, hence the need for this study. This article presents findings from the exploration and description of queer individuals’ opinions of proposed solutions for improving SRHSN in Gauteng Province, South Africa, recognizing the crucial need to prioritize marginalized viewpoints when it comes to healthcare discussions, policy, and decision-making to ensure more inclusive and efficient services.

## 2 Materials and methods

### 2.1 Study design

We performed an explorative-descriptive qualitative study to better understand and identify answers to the issues queer people encounter when promoting SRHSN in Gauteng Province, South Africa. Before the start of the study, the protocol was reviewed by various research committees at Sefako Makgatho Health Sciences University (SMU), including the Department of Public Health Research Committee (DPHRC) and the School of Healthcare Sciences Research Committee (SHSRC), before being approved by the Sefako Makgatho Health Sciences Research Ethics Committee (SMUREC): Protocol number (SMUREC/H/291/2023:PG). The empirical study began in November 2023 and ended in December 2023.

### 2.2 Study setting

Considering the difficulties of easily finding queer individuals, we carried out our research at a queer-inclusive NGO clinic in Gauteng Province, South Africa. The research was done at two study sites: Pretoria and Kempton Park. This inclusive healthcare institution provides free sexual healthcare to homosexual, bisexual, queer, and men who have sex with men (MSM).

### 2.3 Procedures

We recruited 22 queer individuals who attended and used the services. Due to population restrictions and a lack of accurate data, all 22 queer individuals were sampled via RDS. We began by interviewing queer individuals who work at the two branches and then asked them to refer additional participants they know who might be interested. All interested queer individuals were solicited during their consultations the day before data collection, and those who consented were paid 250ZAR for transportation costs. Participants provided a verbal and written agreement form before data collection after outlining the study’s purpose, research questions, and objectives. The written consent form included further details about the study such as the purpose, their rights of voluntary participation, and demographic data without a column for writing their names, thus ensuring that no confidential information is captured to ensure the privacy of the participants.

### 2.4 Participants

Our study included all queer individuals aged 18 and older in the study settings. Our study eliminated those individuals who did not self-identify as queer, those who did not consent, and participants under the age of 18 years. The interviews were conducted in English as the medium language; however, participants were able to react in their native language, and the most usually responding languages were Isizulu and Sesotho, which were then translated into English.

### 2.5 Conduct of the interview and instruments

For this study, we collected data through semi-structured interviews and a semi-structured interview guide. Our semi-structured interview guide was divided into sections, including participant demographics, generic SRHSN questions, and study-specific questions. The interview guide was derived from prior peer-reviewed and published studies (Wanyenze et al., 2016; Müller, 2017; Mkhize and Maharaj, 2021; Kurebwa, 2022; Seretlo and Mokgatle, 2022, 2023). Interview questions were arranged in groups, with open-ended questions supplemented by probing questions, and each interview lasted between 30 min to an hour. Furthermore, we employed a digital audio recorder to record an interview session after obtaining consent from the participants, which was transcribed verbatim after the semi-structured interviews were concluded. Questions that participants were asked included the following: *“In your view, what do you think can be done to improve the current SRHSN for queer individuals?” “What could be done to make access and utilization of SRHSN easier?” “Based on the challenges that you experience at the healthcare facilities; how can those challenges be addressed?*” These questions are attached in the Supplementary Figure S1. All 22 queer individuals answered all the three groups of interview questions. Data were collected by the principal researcher and a research assistant. The principal researcher is a lecturer and a qualitative researcher; however, refresher training was received during the Department of Public Health Winter School, and training was offered to the research assistant by the principal researcher before the data collection. Further support and regular feedback were provided to the research assistant after data collection. Privacy and confidentiality were maintained by using pseudo names for participants, that is, giving them a number based on the interview, (P1, P2, etc.). Data saturation was reached at the 19th interview session, and the principal researcher continued with three additional interviews to confirm the saturation.

### 2.6 Thematic content analysis

We followed TCA to analyze our data while applying four interrelated steps suggested by Ravindran (2019). The first step was to prepare our data where the principal researcher commenced with the translation of all Isizulu and Sesotho data into English using MS Office 365 and working together with an independent transcriber. All transcripts were cleaned, edited, and corrected without losing the meaning intended by the participants. The second step followed where the principal researcher read all transcripts in correspondence with recorded audios and labeled them according to participant’s numbers. The third step was the creation of an initial coding as a form of codebook in an NVivo release 14.23.2 (Weng et al., 2023), QSR International. The principal researcher grouped all the similar codes together and gave them clear meanings, working together with the supervisor who acted as an independent coder to refine the codes. The last step included the development of themes and interpretations to give more concise meanings to the categories that were labeled final themes in NVivo 14. A series of themes and sub-themes were developed and validated using verbatim quotes.

## 3 Results

### 3.1 Pilot study

Three queer participants were recruited to test the semi-structured interview guide in early November 2023. The pilot study was conducted at the Pretoria branch before initiating the main study. Participants for the pilot study were recruited in the same way as the participants in the main study. The principal researcher transcribed the recorded audio and evaluated three transcripts with NVivo 14 software to verify the codes and themes that emerged, verifying that the interview guide outcome was aligned with the intention of the study. Following a meeting between the principal researcher and the main supervisor who acted as a peer reviewer to examine the pilot study data, the semi-structured interview guide was revised twice before being finalized for use in the entirety of the research. Minor updates to the semi-structured interview guide, such as sexual orientation in the demographic section and more probing questions, were implemented. No questions were excluded from the semi-structured interview guide. The pilot research results were eliminated from the main study to avoid inconsistency and misrepresentation of the participants as the initial interview guide was amended twice before the main study; therefore, the results were going to be inconsistent and not representing the same questions, especially probing questions.

### 3.2 Demographics

Our study included 22 queer participants (refer to Supplementary Table S1), whereby under gender identities, we had 19 participants identified as male and 3 as female. Within these gender identities, participants also identified themselves under the following queer classification: gay people accounted for eight, transgender women for seven, lesbians for three, and both MSM and bisexuals for two. When questioned about their marital status, all queer participants said that they were single. Many of the participants had tertiary level education, with various credentials, totaling 12, whereas the minority had secondary level education, specifically matric, totaling 10. Most of the queer participants, specifically 10, completed matric, subsequently 4 obtained a diploma, 3 obtained higher certificates and bachelor’s degrees, and 1 attained certificates and a post-graduate diploma.

### 3.3 Thematic findings

Refer to Supplementary Table S2 for a summary of themes.

#### 3.3.1 Creation of healthcare equity for queer individuals

The importance of developing a healthcare sector that is equal and inclusive for queer individuals emerged strongly from the participants’ feedback. In addition, participants stated that more queer healthcare services should be established at a faster pace and that the reintroduction of school healthcare services to improve health literacy for queer matters among teachers and learners be prioritized. Participants highlighted that the equalization of queer healthcare services throughout different healthcare sectors is required.

#### 3.3.2 Accelerate queer healthcare services

The majority of queer participants suggested that queer healthcare services should be prioritized, and implementation accelerated to ensure their wellbeing and that their needs are met. In addition, issues of long waiting lists and waiting for basic services were stated to be one of the things that should be avoided and healthcare facilities to ensure that long processes are shortened.

“It took me two years to be able to start receiving hormonal therapy, also here at this clinic, because in government I was in a waiting list since then, I would advise that they should try to avoid keeping us waiting for nothing and for long, like again at government for the surgery, you see that one, it is crazy, it’s even worse because like it’s crazy and you can’t be on the waiting list, you wait for a doctor, then psychologists, before a doctor can deem you fit that okay now you are so ready for surgery, Yoh!!” (P11, transgender woman).

#### 3.3.3 Build and expand more clinics for queer individuals

The majority of queer respondents put forward that government should work together with funders and NGOs to develop additional clinics around Gauteng Province that would cater for queer healthcare needs and services around different areas in the province. Other participants compared NGO clinics with public clinics and recommended that if the government cannot build new clinics, they should at least ensure that the public clinics are inclusive, similar to selected NGO clinics.

“So, I personally, I think that we need more places like this, yah, we need more organizations whereby government will fund them and cater specifically for us. I know they will say that we are not a huge number and stuff, but okay even in that government-clinic then, let us have a portion whereby that would be referred as LGBTI alone and we have people who are conscious, like people who are trained or people who live that lifestyle who are qualified” (P15, transgender woman).

“I don’t know what is it the government that must do this but there just has to be-you know if there was a clinic specifically for us, not just lesbians but even gay men, the queer community, like the LGBTQI, if there was a clinic specifically or like in a clinic a unit that is dedicated to us that said okay for conceiving there’s section whatever at whatever clinic or whatever hospital where we offer sperms or whatever there’s a list of sperm donations, you just come, uhm they give you counselling basically” (P22, Lesbian).

#### 3.3.4 Reinstate school healthcare

Predominantly, queer participants advocated for the reintroduction of school healthcare services whereby HCPs would teach, share, disseminate, and inform learners, teachers, school governing bodies, and parents about gender identity. Queer participants believed that this information would help to address the issues of gender confusion, bullying at schools due to different sexuality and gender preferences, and prevention of sexual illnesses and will influence the school policies thus protecting young, vulnerable, queer learners.

“So, I feel and think that people need to be educated in schools, especially like young kids today so that when they grow up, they know that like I’m a queer person, if ever like I want to be like sexually active I have to do these steps and all of that” (P2, gay).

“Let me say like queer kids, I even say teenagers from the age of say like 15 upwards, already they are engaging in sexual acts, and they don’t know the STIs. They only like learned about-I’m not going to lie, in high school they taught us about HIV and STIs I didn’t care, so if school health nurses should be brought back to teach them about protecting themselves because you can’t stop them from having sex, but you can teach them about protecting themselves, seeking medical attention” (P7, gay).

#### 3.3.5 Standardizing care through all healthcare facilities

Most queer participants proposed that uniform and consistent care across the different healthcare facilities, whether private or public, should be ensured and implemented. For example, participants showed that the hormonal services they receive at NGO clinics are not the same as the ones they requested at the public clinics.

“Sometime last year, I went to a public clinic I was asking about hormones, I have asked them if they do have them or not, nurses there said they do have them, but they called it Premarin, I was confused because here we use Estrofem so I would suggest that they should at least have same medications so that when I short of my hormones I can easily go to the clinic without wasting transportation money” (P11, transgender woman).

### 3.4 Empowering and supporting HCPs to enhance skills, knowledge, and expertise

Lack of queer skills, knowledge, and expertise among HCPs became apparent among queer participants as a vast majority of them put forward a need for enriching and guiding HCPs with queer-related healthcare matters. In addition, some queer participants recommended that HCPs should be encouraged to be sympathetic, empathic, and sensitized toward them. Most importantly, queer participants suggested hiring queer individuals across healthcare facilities with a belief that it will enable access and usability of healthcare services by queer.

#### 3.4.1 Improve and provide queer-related training to HCPs

An increased need for HCPs training was recommended by queer participants. Some queer participants indicated that it is the responsibility of the Department of Health to ensure that all HCPs are trained, and some suggested the use of queer activists to assist in training HCPs through workshops and seminars. In addition, queer participants indicated that if HCPs are trained, there would be less judgment rather than an increased understanding of queer patients’ needs.

“I believe people should be educated, like especially, when you go to a public clinic or a public hospital which is like for government. At least have some workshops which your staff, nurses to know how to deal with queer people, you will see us going to public clinics and hospitals without fear” (P6, bisexual).

“I feel like the health service workers should be trained on how to treat us because of obviously now ever since that there are hormones that are involved our emotions are very high sometimes and then like we are very short tempered, emotional, and whatsoever, I’m very emotional. So now I believe that they do not even accommodate us when it comes to that you know” (P13, transgender woman).

#### 3.4.2 Hiring of queer HCPs

The majority of queer voices conveyed that employing queer individuals, who focus and deal with their matters, could be one of the solutions to prejudice, discomfort, and changing of the negative attitude of HCPs toward them, thus improving accessibility and usability of SRHSN by queer individuals in the public healthcare facilities.

“I think the only thing that might be done in public hospital, I think they should hire more queer health professionals, like two maybe-like two or three will make a difference. Those people are going to be-they’re going to teach; they’re going to teach. Like if ever, imagine like its 50 nurses, all of them are straight they don’t know anything about queer except of you trying to be a girl, you trying to do one, two, three, but whenever we hire queer health professionals or nurses, queer nurses, they’re going to use that platform to teach. Like if ever you are handling a queer person, you don’t ask this-certain questions, you treat them like this, you treat them like how you treat every patient” (P7, gay).

“I think maybe they should employ two gays or two LGBTQ+ member to be there, you know, maybe they can’t be that judgmental because they’ll have always taught them-each and every day they will be seeing that person as their colleague too” (P16, gay).

#### 3.4.3 Motivate HCPs to emphasize and synthesis

The consensus among queer participants leaned toward a need for encouraging HCPs to become more considerate and harmonized whenever talking and rendering healthcare services to queer patients.

“I think more sensitization, I think that’s the only, well not only, but that’s what comes to mind. So, if the people that are accessing the services are sensitized and the people that are providing the services are sensitized, I think that would be a little bit better. So, I think the staff has received sensitization so that’s why there is less or no challenges, or you know, but maybe clients. So, you meet somebody on the street who might need the service but, you know, just because it’s a gay-it’s seemed as a gay clinic, or its-don’t know how to put it but, it’s seemed as everyone who’s gay goes there then they would not want to. But if that person has been sensitized, I don’t think they would have that assumption” (P5, gay).

“I think, how can I say it? if the-let’s say government officials the ones who works at the clinics and stuff, they could be taught to sensitize things because we are very sensitive because every time a person will call you names you will automatically retaliate, so if they could just speak from a sense of sensitivity and also know that I should approach this person with a sense of sensitivity, that I should not just speak however I want” (P12, transgender woman).

### 3.5 Raise awareness on queer-related issues

Increasing awareness of queer healthcare matters was proposed by the majority of queer participants stating that information regarding queer existence should be shared with different campaigns and social media sites. In addition, some queer participants suggested that the selected NGO clinic, and other queer NGOs, should be made public so that all queer individuals can access and utilize them, thus improving their health and wellbeing.

#### 3.5.1 Organize campaigns

Different types of campaign locations, such as malls, hospitals, community halls, and schools, were pointed out and recommended as some of the ways that could address stigma and discrimination, thus enhancing the sexual-reproductive health wellbeing of queer individuals across the Gauteng Province.

“I really think that if we can get like a lot of activists who can have different engagements. They talk to people using these things, what do you call them? Oh yeah, campaigns, not just only at the streets, but go to the mall, have campaigns, tell people, show yourself, teach them about their sexual health. I feel like that’s how you can thingy-yah. Many queers will be free and feel safe” (P18, transgender woman).

“I guess more awareness, more maybe like campaign for awareness, I mean that used to happen but doesn’t happen anymore. I mean you know what I mean like-and even in that it’s like have somebody who is not even in the healthcare facilities come to that awareness and maybe speak about their journey in not that it’s a journey it’s you but it’s like the way in which maybe they became very comfortable around them being homosexual and stuff like awareness are really important for everybody, teach people about the importance of condom use and also share what we needs for our reproductive health” (P21, lesbian).

#### 3.5.2 Announce on social media platforms

Online social networks were recommended as some of the ways that sexual and reproductive healthcare needs and services can be met and be inclusive. Queer participants believed that every person in this era is using different media platforms such as television, Facebook, Instagram, and Twitter. If information is disseminated using different media platforms, it will reach a lot of queer individuals who never had information, thus improving inclusivity and their sexual and reproductive health.

“So, if ever maybe we government introduce like educational programs that appear on television, you know people would be like I want to see more of this, and the more they are hooked, that’s the more they learn. Imagine a program that speaks about sexual diseases that affects queer people or things like how queer people can protect themselves” (P12, transgender woman).

“You know, there was a programme that focused on queer health, like Soul Buddy, so if we can have like something similar can be introduced but on social media platforms like Twitter, your Facebook, and your Instagram, it would be nice and helpful, that’s my advice, you know. To have different topics every now and then so that maybe if you discover that there’s a new illness or whatever you can just put up did you know about whatever, and then it shows you, while you’re scrolling through those social networks” (P20, lesbian).

#### 3.5.3 Publicize NGOs supporting the queer community

A significant number of queer individuals proposed that existing queer NGOs, such as the selected NGO clinic, should be advertised and made known to the public. Queer participants trusted that if these places are publicized, queer individuals will be able to obtain and use different sexual and reproductive services such as PrEP and HIV testing.

“Honestly speaking I think uhm because a lot of gay people-let me just speak-let me just say gay people don’t even know that this place exists so if ever like this place can be marketed in like uhm social media and let people know, spread the word about it. It will make gay people feel more responsible because a gay person will literally be so comfortable coming here than going to a uhm government hospital and dealing with the nurses’ bad attitudes and whatever. But another important thing is they will be able to access different sexual services such as PrEP, HIV testing, you see. Because a lot of people they can, even if you get sick, for an example, maybe I have a sore on my private part, I want to seek medical attention, it’s going to be-I’m just going to be like let me just go to the pharmacy and get maybe antibiotics, it’s going to die down, I don’t want to go to uhm the clinic because I don’t want to be judged” (P7, transgender woman).

### 3.6 Different stakeholders’ involvement

Most queer participants recommended that HCPs are not the only group of professionals and people that need to be involved in ensuring that SRHSN for queer individuals is inclusive. The majority of queer participants further suggested active engagement, participation, and involvement of different key players and relevant parties such as police officers so that queer participants are included in their SRHSN thus reducing judgment.

#### 3.6.1 Community members and government officials

The importance of involving community members and governmental officials is one of the strategies that could improve sexual and reproductive health for queer people. Queer participants stated that police officer’s attitudes hinder them from reaching and utilizing SRHSN such as rape kits.

“Most of us when we are raped, we are scared of going to the police station, they do not want to help us and they call us with names, I suggest that all government staff, whether its clinics, hospitals, police stations everywhere, home affairs, everything, let them be trained, understand, on how to address and service the LGBTI community. Also, educate the entire community” (P11, transgender woman).

#### 3.6.2 Political leaders

Policymakers and authorities’ duties should be to promote inclusivity, thus achieving queer SRHSN. Queer respondents recommended that policymakers and authorities are the ones to lead the change and be role models.

“Uhm, I think, I think this should be addressed especially when it comes to the President, should you know, at least listen to us so that we can tell him like the things that we need and whatever we are going through when it comes to public spaces so yah, I think so” (P13, transgender woman).

#### 3.6.3 Queer individuals themselves

Queer-identifying individual’s responsibility to take full care of themselves was suggested by queer participants themselves. Queer participants alluded to the fact that if they take complete care of themselves, many sexual and reproductive health issues will be resolved.

“I think LGBTQI+ people should take a big responsibility for their health, So, like how do I first take responsibility for my life, one, two for my sexual health because I mean HIV is up there and STIs, so how do I become responsible, you know, do I use a condom every time when I have sex. If I’m on Grindr, how am I taking precautions, not only keeping my life safe but also sexually, how do I make sure that I protect myself, do I get on PrEP, you know. Also, if-to such an extent, the community, how do I make sure that I’m safe. Do I-when I go out do I go out alone, you know, with these men that I know that would-they would want a beer, or they would want money, or they would want cigarettes, or they would want to have sex with me. How do I make sure that I protect myself? We-sensitization and as individuals, how do we take care of ourselves and protect ourselves, either physically, sexually, mentally, and otherwise. How do we take on us and say I’m going to take care of myself because other people that have been sort of like in this journey or are out there to really-unfortunately they are overstretched so they cannot reach this point but how do I carry myself from this point to make sure that I’m safe” (P5, gay).

## 4 Discussion

Our study aimed at exploring and describing queer individuals’ perceptions and solutions to promote SRHSN in Gauteng Province, South Africa. Our research revealed the critical need for the creation of an equal and inclusive healthcare system for queer individuals. Our findings support Bennett et al. (2017) advice that more work needs to be done to promote acceptance of diversity and the inclusion of lesbian, homosexual, bisexual, and transgender families in the design, development, evaluation, and access to early parenting services. Again, our findings support Mulemfo et al. (2023) conclusion that gender diversity, inclusion, and sensitivity when it comes to healthcare delivery, as well as specific LGBTQI+ training for healthcare providers, are critical elements in making sure LGBTQI+ individuals’ access to quality HIV management support services. Our study concurs with the recommendations outlined in a study by Furness et al. (2020) that creating more inclusive and culturally supportive workplaces can boost LGBT participation in healthcare, ultimately leading to improved general wellbeing and health. Considering these results, it is essential that policymakers, governments, and external healthcare stakeholders need to join hands together to ensure the provision of the same and comprehensive healthcare services as provided to heterosexual clients. These could include services such as the utilization of correct pronouns, offering gender-neutral wards and bathrooms, and continuous training and workshops to the HCPs.

Furthermore, there was a need for the restoration of school healthcare services to improve health literacy regarding queer issues among both teachers and learners. Our research aligns closely with several studies that showed that school-based interventions can improve the wellbeing of queer learners (Kaczkowski et al., 2022; Kesler et al., 2023; McDermott et al., 2023). We understand that these studies were not entirely about queer SRHSN, but we interpret this to mean that school healthcare support is important to improve the overall wellbeing of queer learners. For example, the literature showed that context factors such as a ‘whole-school approach’ and ‘collaborative leadership’ were crucial to the delivery of successful interventions, and there was potential to improve mental health (McDermott et al., 2023). Furthermore, consistent with existing literature, our study concurs that multifaceted LGBTQ-supportive school policies and practices can improve sexual health outcomes among both LGB and heterosexual students, such as less sexual risk behaviors such as having fewer sexual partners, testing HIV negative, and utilization of condoms during sexual intercourse (Kaczkowski et al., 2022). Our findings substantiate the assertions put forward by Kesler et al. (2023) that inclusive comprehensive sexual health education programs such as High School Family Life and Sexual Health (FLASH) can help promote better educational environments for all youth by decreasing opinions that result in harassment, assault, and being victimized. FLASH is a comprehensive sexual health education curriculum developed and maintained by a county public health department (Kesler et al., 2023). Furthermore, it is a public health strategy for classroom settings, with the specific behavior change goals of preventing unintended pregnancy, preventing STIs, and preventing the perpetration of sexual violence (Kesler et al., 2023). Given this information, it is vital that policymakers consider incorporating and amending the existing South African integrated school health policy to include matters relating to queer individuals as stated above. In addition, policymakers could involve existing NGOs to help with amendment thus ensuring inclusivity at the school level and enhancing health literacy among teachers and learners.

Queer participants emphasized the significance of HCPs obtaining specialized training in queer healthcare. We can be confident that if HCPs are educated on queer-related health issues, there will be a significant improvement in knowledge, attitude, and/or practice after training (Sekoni et al., 2017). Overall, it appears that whenever queer education curriculum is conducted among undergraduate medical students and training HCPs in queer competencies, their attitudes change and improve post-training and their knowledge toward queer people increases (Sawning et al., 2017; Donisi et al., 2020; Wahlen et al., 2020; Yu et al., 2023). It was suggested that the Department of Health in South Africa should take responsibility for ensuring that all HCPs receive proper training and that queer activists should be involved to provide workshops and seminars for HCPs. Our findings are congruent with a study by Luvuno et al. (2019), which found training should be provided during basic education and in-service to enhance healthcare workers’ abilities, attitudes toward LGBT patients, and therefore access. In general, our study is similar to one conducted by Nicol et al. (2019), who, while not focusing on queer persons or SRHSN, found evidence that diverse and wide training interventions could increase HCPs’ knowledge, skills, and competency in vaccination data management. Our findings confirm Hunt et al. (2019) conclusion that more effective training strategies based on behavior change techniques are required. In light of these findings, it is crucial that higher education institutions and healthcare facilities establish a curriculum that focuses on queer health matters for undergraduate, post-graduate, and HCPs who are already at the workplace. Furthermore, these could be attained by developing compulsory detailed diversity and inclusive training for all HCPs as a form of in-service training, workshops, and professional development programs.

Participants agreed that more awareness should be raised regarding gay healthcare issues. The recommendations included promoting awareness about queer existence via campaigns and social media platforms. Our research found that the majority of literature from various spheres and areas emphasizes the need to raise LGBTQ+ understanding. For example, Cooper et al. (2020) recommend that biological educators and researchers raise awareness of LGBTQ+ identities and start discussions about converting biology educational environments and the larger academic biology community to be more inclusive of LGBTQ+ people. Again, Hull et al. (2017) found that exposure to the campaign significantly correlated with acceptance among gay men, indicating effective communication targeting. The association between exposure and acceptance of gay men was significantly influenced by views regarding gay men, views of societal acceptance, and views of the influence of stigma on gay men, yet not by the rejection of stereotypes (Hull et al., 2017). Based on this evidence, it is necessary that healthcare facilities commence with the introduction of queer health days same as any other health issues to ensure awareness among HCPs and supporting staff within the institutions. These activities could be done in collaboration with existing queer NGOs and community members so that knowledge is shared thus achieving an understanding of queer matters.

In terms of using social media to raise awareness, several studies agree with our findings that it promotes support and development, general educational goals, and the acquisition of LGBTQ+-specific material (Craig et al., 2021). Chan (2023) found that incorporating LGBT social media into social routines was associated with reduced levels of internalized judgment and a higher degree of community connectivity, both of which were linked to improved wellbeing. Our findings are consistent with those of Garcia et al. (2016), who discovered that peer networks are critical components in improving the effectiveness of combined prevention among Black men who sleep with other men (BMSM) by reducing internalized homophobia and cultivating emotional support from others. Furthermore, our findings confirm the findings of Berger et al. (2021) who demonstrated that it can aid queer individuals who feel lonely or victimized by providing information on sex and sexual knowledge. However, Berger et al. (2022) study on the use of social media on the health wellbeing of LGBTQ youth discovered that mental health difficulties associated with social media use were caused by discrimination, victimization, and policies that failed to account for changing identities. The same study found that social media could benefit LGBTQ youths’ mental health and wellbeing through peers’ relationships, managing their identities, and social assistance, although the conclusions were limited by insufficient proof (Berger et al., 2022). The utilization of different social media platforms should be considered and prioritized to be the means of communication to ensure that awareness is raised.

Furthermore, it was advised that groups, such as the selected NGO clinic and other queer services NGOs, be made more accessible to the public to enhance the health and wellbeing of queer individuals. Our findings are consistent with the agreement in the field of social advertising that many LGBT concerns can be addressed (Chauhan and Shukla, 2016); in addition, the respondents believe that it would instill a sense of bravery and authority in the LGBT community. They went on to say that they contend that commercials depicting social issues should go beyond traditional product advertisements to encourage societal change in society’s perspective regarding these issues and eliminate narrow-minded ways of thinking (Chauhan and Shukla, 2016). Chauhan and Shukla (2016) argue that commercials help society achieve sexual freedom. Our findings closely align with the conclusions drawn by Fauzi and Setyaningrum (2016) that the implementation of NGO support on ensuring excellent healthcare available may enhance the wellbeing of LGBT people and minimize prejudice itself among the community through social support engagement in intrapersonal relationships (acceptance of oneself and enhanced mental wellness), interpersonal (a decreased loneliness and relationships), society (reciprocation, decreased stigma, and prejudice), and the structure (housing and work). Healthcare facilities ensure that queer-related services are rendered and available thus ensuring inclusivity and good health for all. With these observations in mind, it is imperative that all public healthcare facilities should improve their services to ensure that SRHSN of queer individuals is achieved to improve their wellbeing and promote their health.

Importantly, participants emphasized that healthcare practitioners are not the only stakeholders in safeguarding queer individuals’ SRHSN. The inclusion of varied experts and stakeholders in many settings was judged critical to attaining complete and inclusive healthcare for queer individuals. A literature study showed that training professionals in different fields improve the attitudes toward LGBT+ individuals, as shown in a study by Anderson et al. (2020) which showed that participants who attended training on LGBTQ and were working in a recreational collegiate aquatic setting scored higher on average in both attitudinal and competency-based measures. Our findings support an argument by Weng et al. (2023) who showed that when compared to conservative chief executive officers (CEOs), liberal CEOs will be more likely to support LGBT employees by implementing LGBT-friendly policies. Finally, Jones (2019) interviewed key informants who identified how such contributions have a strong human rights emphasis, furthering post-colonial resistance to simplistic gender and sexuality classification schema imposed via imperial colonizing dynamics.

### 4.1 Future directions

Moving forward, future studies must prioritize the inclusion of queer individuals to ensure that their health needs are served properly, even outside the scope of research efforts. By amplifying and adopting queer voices’ proposals, we can strive toward a healthcare system that is truly empathetic, accommodating, and equitable for all.

Furthermore, the research findings could be used to advocate for policy changes that promote queer rights and improve SRHSN services. Collaborating with lawmakers, advocacy groups, and community organizations can help to advance legislative reforms and institutional improvements that emphasize the needs of queer individuals. Community engagement and empowerment measures should be used to involve queer people in advocacy efforts and make their views heard. This could include the creation of support networks, peer education programs, and community-led initiatives to reduce healthcare inequities. Finally, future directions ought to emphasize queer individuals’ active participation in shaping research agendas and interventions, fostering collaboration among researchers, policymakers, healthcare providers, and community stakeholders to create meaningful and long-term change in SRHSN services.

### 4.2 Strengths and limitations

#### 4.2.1 Strengths

Our study used an explorative-descriptive methodology, which allowed us to gain a thorough grasp of queer people’s experiences and viewpoints inside the healthcare system. RDS is a regularly utilized strategy for reaching out to hidden communities, such as queer individuals, and it can help to assure sample diversity. Semi-structured interviews provide flexibility in data gathering, allowing participants to comment on their experiences in their own words. Allowing participants to respond in their preferred language improves accessibility and may result in more real responses. Thematic analysis is an effective tool for discovering patterns and themes in qualitative data and using NVivo can improve the rigor and efficiency of the analysis process.

#### 4.2.2 Limitations

Despite the use of RDS, there is a possibility of sample bias because participants were recruited from a single NGO in Gauteng Province, South Africa, which may not represent all queer individuals in the region. The sample size was one of our study limitations as the study focused on a single NGO and due to the scarcity of queer individuals. While efforts were taken to translate interviews done in Isizulu and Sesotho into English, nuances and meanings may have been lost during translation, thereby affecting the data’s inherent meaning. The findings may not be generalizable beyond the unique setting of Gauteng Province, South Africa, and may not reflect the experiences of queer people from other areas or nations.

### 4.3 Conclusion

Our research emphasizes the valuable viewpoints and recommendations made by queer people regarding sexual and reproductive healthcare services in the Gauteng Province, South Africa. We conducted semi-structured interviews with 22 participants in English, Isizulu, and Sesotho, using respondent-driven sampling inside the NGO clinic, to explore their perceptions and solutions. These findings emphasize the importance of including queer voices in discussions about sexual and reproductive health, as they provide unique and critical ideas that challenge existing conventions and pave the way for a more inclusive and equitable healthcare system.

## Data availability statement

The raw data supporting the conclusions of this article will be made available by the authors, without undue reservation.

## Ethics statement

The studies involving humans were approved by Sefako Makgatho Health Sciences Research Ethics Committee (SMUREC): Protocol number (SMUREC/H/291/2023:PG). The studies were conducted in accordance with the local legislation and institutional requirements. The participants provided their written informed consent to participate in this study.

## Author contributions

RS: Conceptualization, Data curation, Formal analysis, Funding acquisition, Investigation, Methodology, Software, Writing – original draft, Writing – review & editing. MM: Formal analysis, Methodology, Supervision, Validation, Visualization, Writing – review & editing. HS: Methodology, Supervision, Validation, Visualization, Writing – review & editing.
