# Supplementary material for: Breaking challenges: queer perspectives on solutions to establish inclusive sexual-reproductive healthcare in Gauteng Province, South Africa
Source: Front Sociol. 2024 Aug 6;9:1406265. doi: 10.3389/fsoc.2024.1406265 (PMC11334262; doi:10.3389/fsoc.2024.1406265)
Supplement: Supplementary file 1 [file Data_Sheet_1.docx]

Supplementary Material

# Supplementary Tables

## Supplementary Tables

**TABLE 1: Demographics of queer individuals.**

| **Demographics** | **Frequency**  ***n (=22)*** |
| --- | --- |
| **Age, Mean (SD)** | **25.9 (.89)** |
| **Gender identification *n (%)*** |  |
| *Male* | **19 (86.36)** |
| *Female* | **3 (13.64)** |
| **Respondents identifying as queer *n (%)*** |  |
| *Lesbian* | **3 (13.64)** |
| *Gay* | **8 (36.36)** |
| *Bisexual* | **2 (9.09)** |
| *Transgender woman* | **7 (31.82)** |
| *MSM* | **2 (9.09)** |
| **Marital status *n (%)*** |  |
| *Single* | **22 (100)** |
| *Married* | **0** |
| **Education level *n (%)*** |  |
| *No formal education* | **0** |
| *Primary* | **0** |
| *Secondary* | **10 (45.45)** |
| *Tertiary* | **12 (54.55)** |
| **Highest qualifications *n* (%)** |  |
| *Matric* | **10 (45.45)** |
| *Certificate* | **1 (4.55)** |
| *Higher certificate* | **3 (13.64)** |
| *Diploma* | **4 (18.18)** |
| *Bachelor’s degree* | **3 (13.64)** |
| *Post graduate diploma* | **1 (4.55)** |
| *Honors degree* | **0** |
| *Master’s degree* | **0** |

**TABLE 2: Queer’s suggestions on improving SRHSN thematic analysis.**

| **THEMES** | **SUB-THEMES** |
| --- | --- |
| Creation of healthcare equity for queer Individuals. | - Build and expand more clinics for queer individuals. - Reinstate school healthcare. - Establish queer-specific clinics. - Standardizing care through all healthcare facilities. - Accelerate queer healthcare services. |
| Empowering and supporting HCPs to enhance skills, knowledge, and expertise. | - Improve and provide queer-related training to HCPs. - Hiring of queer HCPs. - Motivate HCPs to emphasize and synthesize. |
| Raise awareness on queer-related issues. | - Organize campaigns. - Announce in social media platforms. - Publicize NGOs supporting the queer community. |
| Different stakeholders’ involvement. | - Political leaders. - Community members and government officials. - Queer individuals themselves. |

**Figure 1: An interview guide**

**Section A: Introduction & Demographic data**

**Introduction**

Thank you for agreeing to participate in this interview. My name is Raikane James Seretlo, from the School of Public Health in Sefako Makgatho Health Sciences University. I am talking to self-identified queer individuals who are receiving and utilizing sexual-productive healthcare services around district hospitals/NGOs in Johannesburg and Tshwane, I would like to explore and describe queer individuals’ perceptions and solutions for promoting sexual-reproductive healthcare services and needs (SRHSN) in Gauteng Province, South Africa.

I will be using a digital recorder so that I can transcribe the information provided by you after the interview. I would like to assure you that all the information gathered in this room will be kept confidential and your personal identity will be protected at all times. Feel free to tell me if you are not comfortable with any of the questions, but it would be appreciated if you could answer as many of the questions as possible. Remember that participation is voluntary, and you are free to withdraw from the study if you choose to without consequences.

|  | **Demographic Information:** |  |
| --- | --- | --- |
| 1 | Place of data collection |  |
| 2 | Age |  |
| 3 | Gender identification |  |
| 4 | Sexual orientation/ Respondents identifying as queer |  |
| 5 | Marital status |  |
| 6 | Highest qualification |  |

**Section B:** Semi structured questions

**General questions:**

1. **What do you understand the concept sexual-reproductive healthcare services and needs?**

- **Probes:**
- Can you tell me what the concept sexual-reproductive healthcare services and needs mean to you?
- Give examples of sexual-reproductive healthcare services and needs you commonly access/utilize?

**Main questions & probes:**

1. In your view, what do you think can be done to improve the current sexual-reproductive healthcare services and needs for queer individuals?
2. What could be done to make access and utilization of SRHSN easier?
3. Based on the challenges that you experience at the healthcare facilities; how can those challenges be addressed?

**
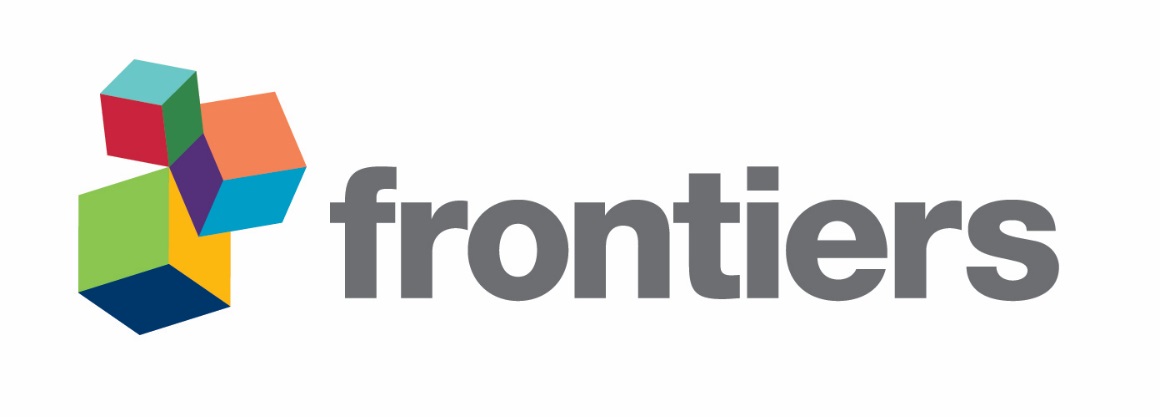
**
